# Acellular bovine pericardium as a biological dressing for treatment of cutaneous wounds of the distal limb in donkeys (Equus Asinus)

**DOI:** 10.1007/s11259-022-10014-9

**Published:** 2022-11-03

**Authors:** Mohammed Albahrawy, Khaled Abouelnasr, Esam Mosbah, Adel Zaghloul, Marwa Abass

**Affiliations:** Department of Surgery, Anesthesiology, and Radiology, Faculty of Veterinary Medicine, Mansoura University, 35516 Mansoura, Egypt

**Keywords:** Donkeys, Bovine pericardium, Distal limb wounds

## Abstract

This research was performed to determine the impact of repeated topical dressing with acellular bovine pericardium (ABP) on healing distal limb wounds in donkeys. Twelve male clinically healthy donkeys were subjected to general anesthesia, and full-thickness wounds of six cm^2^ (2 × 3 cm) were created on the middle dorsolateral surface of the metacarpi. Two defects were made on each donkey’s forelimbs; the right limb was considered a control wound, and the left one was considered a treated wound. Moreover, the control wounds were irrigated with saline every three days postoperatively and bandaged with a standard dressing. The treated wounds were covered with ABP dressings. The ABP dressing was reapplied thrice at 7-, 14- and 21-days post-wound induction. In addition, the wound healing process was monitored clinically, histopathologically, and immunohistochemically of tissue as growth factor-β1, epidermal growth factor receptor, and vascular endothelial growth factor. Besides, the gene expression profile of angiogenic and myofibroblastic genes was applied as vascular endothelial growth factor-A, collagen type 3α1, fibroblast growth factor 7, and the transforming growth factor-β1.

The results revealed that the wounds treated with ABP healed more quickly than the control wounds. Additionally, the mean days required for healing were significantly shorter in the ABP-treated wounds (p < 0.05; 69.5 ± 1.6) compared to control wounds (86.3 ± 3). Furthermore, immunohistochemical and gene expression analyses were significantly improved in ABP wounds than in control wounds. In conclusion, ABP is considered a natural biomaterial and promotes the healing of distal limb wounds in donkeys if applied weekly during the first three-week post-wound induction.

## Introduction

Equines frequently suffer from traumatic injuries associated with prolonged healing and are commonly associated with many complications (Wilmink and van Weeren 2004). Achieving proper healing and decreasing associated complications are still challenging for veterinarians (Lawless et al. 2020). The normal wound repair process consists of 3 phases, inflammatory, proliferative, and remodeling (Provost 2018). The proliferative phase begins with the production of granulation tissue, which fills the wound gap, followed by re-epithelialization and wound contraction, named the second healing intention (Sparks et al. 2020). Distal limb wounds are more susceptible to multiple complications due to high tension, lack of supportive soft tissue, diminished blood supply, bony prominence, high motion rate over joints, and the propensity for infection owing to proximity to the ground (Wilmink et al. 2006; Varhus 2014). Optimal wound healing requires avoidance of exuberant granulation tissue, an elevated contraction rate, and accelerated epithelialization (Jørgensen et al. 2021). ABP is a natural, inert, inexpensive, easily obtained biological scaffold material.

Furthermore, the cell removal from the ABP results in a complex mixture of functional and structural proteins forming the extracellular matrix (ECM) (Dong et al. 2009). The effectiveness of its application in full-thickness cutaneous wound healing depends on the architectures and structures of the matrices that may serve as scaffolds for promoting cell proliferation and the formation of granulation tissue (Zajicek et al. 2012; Van Rijn et al. 2016). Previous research has demonstrated the successful use of ABP on the abdominal wall defect reconstruction in dogs, tenorrhaphy and tendon elongation, valves and patch grafts in cardiac surgery in humans, and cutaneous wound healing in rabbits (Anson and Marchand 1996; Van Rijn et al. 2016; AL-Bayati and Hameed 2018). This study aimed to assess the effect of ABP- dressings on the healing of distal limb wounds in donkeys.

## Materials and methods

### Animals

GPower version 3.1.9.7 was used to perform an a priori power analysis to estimate the minimum sample size necessary to verify the research hypothesis. For repeated measures, the sample size needed to obtain 80% power for identifying a medium effect at a significance level of α = 0.05 was N = 12. Consequently, the obtained sample size of N = 12 is sufficient to verify the research hypotheses. Accordingly, twelve healthy male donkeys, ranging in age from 4 ± 1.3 years and weighing 140 ± 42.5 kg, with no blemishes or scars on the metatarsal and metacarpal regions, were included in this study. The donkeys were clinically assessed for evidence of lameness or pain. Fourteen days before the trial, they were given an anthelmintic medicine: Equiveen Paste (0.2 mg/kg Per Os) (Ivermectin Paste, Adwia Company, Egypt). Animals were housed in separate stables and provided a balanced diet.

### Bovine pericardium (BP) sheet preparation

The bovine pericardium was collected immediately after slaughtering the animal from a local abattoir. During transportation, the BP was immersed in phosphate-buffered saline (PBS) (PH = 7.2). The tissue was delicately rinsed with PBS to remove the adhering blood. The BP was manually and mechanically cleaned with dry gauze to remove any fat or connective tissue. The pericardium was decellularized utilizing (4%) ethanol and (0.1%) peracetic acid for two hours, followed by 15 min of cleaning with deionized water and PBS. The produced ABP was kept at 4 °C in PBS containing 1% gentamycin (Gentacure-10, Pharma Swede Co, Egypt) at a volume ratio of 9:1 until usage (Freytes et al. 2008).

## Experimental study

On the operation day, an intravenous catheter was inserted aseptically into the jugular vein of each donkey. All donkeys were given a single injectable dosage of an antibiotic containing penicillin (6 mg/kg, Norocillin LA, Norbrook Company, United Kingdom). The anesthetic protocol included; premedication with acepromazine maleate intravenous injection (0.05 mg/kg- Castran 1.5%, Interchemie Company of Holland). After 15 min, xylazine HCL (1 mg/kg- Xylaject 2% - Adwia Company, Egypt) was administered intravenously. Five minutes later, a propofol bolus dose of (2 mg/kg- Pofol 1%, Eimc Company, Dongkook, Korea) was administered intravenously, and anesthesia was maintained with a 0.2 mg/kg/min propofol infusion rate. All donkeys were maintained in the dorsal recumbent position with fully extended forelimbs. The hair of the metacarpal region was clipped circumferentially, shaved, and scrubbed with isopropyl alcohol and iso-Betadine in preparation for aseptic wound induction. A tourniquet was applied around the antebrachium of the limb. A sterile metal rectangular template (3 × 2 cm) was positioned in the middle in aseptically prepared areas. With the aid of a No. 22 scalpel blade, full-thickness wounds were generated on the dorsolateral side of the mid-metacarpi by incising the skin around the rectangular metal template. Hemostasis was accomplished utilizing mechanical pressure as well as tampons. Wounds were created on the forelimbs of every donkey (12 defects per group). Each donkey had an ABP- treated wound on the left limb and a control wound on the right limb.

**Fig. 1:**
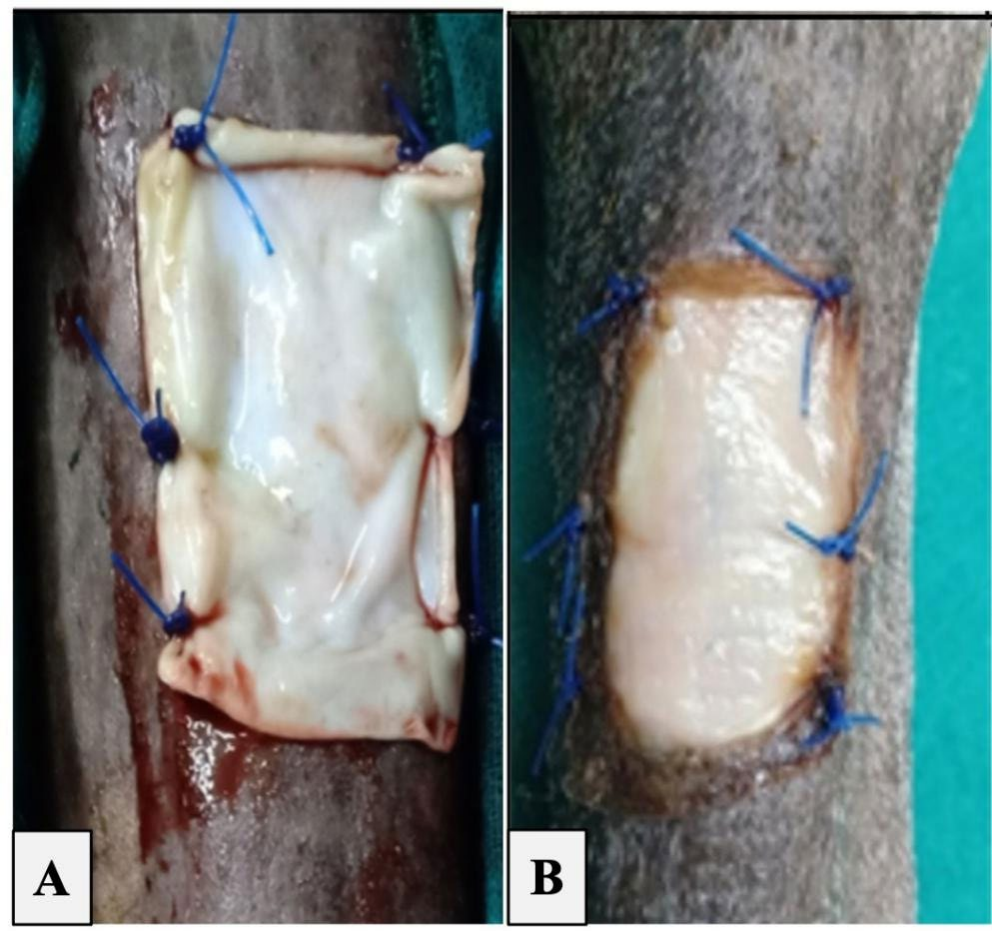
A. fixation of acellular bovine pericardium (ABP) membrane with wound margins. B. One week after application of ABP with brown coloration of its boundaries

In the ABP-treated wounds, the ABP fragment was sliced roughly 0.5 cm larger than the wound and washed for 15 min in a saline-containing penicillin antibiotic solution (Ahmadpour et al. 2018). The wound was subsequently dried between two sterile gauze wipes before suturing ABP (Fig. 1 A). A simple interrupted suture pattern using polypropylene monofilament suture material (Prolene, Ethicon, Inc., Somerville, N.J.) was used during the fixation of ABP dressings. The tourniquet was removed. In control wounds, protective non-adherent dressings were applied to both forelimbs’ wounds. The wounds were wrapped with a standard non-adherent distal limb bandage consisting of 3 layers. The first inner contact layer of the bandage was non-adherent dressing sterile gauze (Derma-Tulle, Gauze Pads, Telfa, 10*15, Dressing Medical Me, Egypt). The second padding layer consisted of a sterile absorbent dressing pad (Surgical pad 10ҳ10cm, Tri M Medical, 10th of Ramadan City, Cairo, Egypt) that was secured with soft, elastic roll cotton (Sof-pan El Mahalla Co., El-Mahalla, Egypt). The third layer was formed of gauze (10 cm, El Mahalla Co., El-Mahalla, Egypt) and elastic adhesive tape (Silk Plast Adhesive Tape 10 cm, Pharmaplast Co, Kafr El-Zayat, Egypt).

## Postoperative care

At the end of the operation, the donkeys were administered butorphanol (50 µg/kg, Torbugesic, Fort Dodge, IA, USA) IV, and donkeys were placed into a padded recovery room. For 3 successive days postoperatively, butorphanol was injected intravenously (50 µg/kg, Nargesic®; ACME Srl, Reggio Emilia, Italy; (Straticò et al. 2021). The first dose was administered 4 h following wound induction. In addition, a single dose of penicillin-based antibiotic (6 mg/kg, Norocillin LA, Norbrook Company, United Kingdom) was injected intramuscularly into all donkeys.

The control wounds were irrigated with saline then the bandages were removed every three days after wound induction (the day that wounds were created was designated as day 0) until the 28 days postoperatively. In the ABP-treated wounds, the ABP dressing was replaced on 7th, 14th, and 21st days. The ABP dressing was removed and resutured standing with local infiltration of lidocaine HCl (Debociane, 2 mg/ml, ADWIC/El-Debeiky) on 7th, 14th, and 21st days postoperatively. Wound margins in both groups were gently cleaned of any exudate with dry, nonsterile gauze sponges and washed with saline.

## Clinical assessment of wound healing

All wounds were evaluated in terms of epithelialization, contraction, and the formation of granulation tissue based on digital photographs immediately taken after inducing wounds until full wound epithelialization (at 1st, 3rd, 5th, 7th, 9th, and 10th weeks post-wound induction). A single-blinded investigator (MA) assessed and examined the wounds. The wound size was measured using the digital caliper. The ratio of healing, epithelialization, and wound contraction was estimated based on the formula of (Karayannopoulou et al. 2015). The percentage of wound contraction = 100 – (wound size at day (x) mm^2^ / wound size at day (0) mm^2^ × 100). While wound epithelialization percentage = size of epithelialization area at day (x) mm^2^ / size of the wound at day (0) mm^2^ × 100. Whereas the percentage of wound healing = 100 – (granulation tissue at day (x) mm^2^ / size of the wound at day (0) mm^2^ × 100). The granulation tissue bed was scored and, if deemed exuberant (i.e., grade 4), resected with a No. 22 scalpel blade to the level of the surrounding epithelium under the anesthetic protocol mentioned earlier. Wounds were considered healed when completely covered by epithelium.

## Histopathological assessment

Wound biopsies were obtained 14 and 42 days after the wound induction under the anesthetic protocol described previously. Under aseptic conditions, samples were collected from the margins of edges containing 2 to 3 mm of normal skin. Samples were placed in neutral-buffered formalin (10%) for 24 h, followed by fixation in paraffin before being stained with Masson trichrome stain as well as hematoxylin-eosin (H&E). A semi-quantitative analysis of histological sections was performed utilizing the scale described by (Vidinský et al. 2006).

## Immunohistochemical assessment

Tissues fixed in paraffin were sliced (4 μm thick) and put on the slides of saline-coated glass. Sections were deparaffinized in xylol and dehydrated in a variety of ethanol concentrations. The antigen was extracted by autoclaving for 10 min at 120^o^C at a pH of 6.0. Endogenous peroxidase activity was inhibited for 10 min with 3% H_2_O_2_. The tissue slices were then treated with primary antibodies against EGFR, FGF, and TGFβ (ready to use, Bio Genex). Following a one-hour incubation at room temperature, (tissue slices) were washed three times with phosphate buffer saline. The tissue slides were incubated at room temperature with anti-rabbit secondary antibodies for 30 min and visualized by incubation with the three diaminobenzidine tetrahydrochloride liquid system (Dako) at room temperature for 5 min. Subsequently, sections were counterstained utilizing hematoxylin.

## Gene expression assessment

Tissue samples were lysed and homogenized utilizing Trizol reagent (Invitrogen, Carlsbad, CA, U.S.A.). With an Implen spectrophotometer, RNA purities and concentrations were examined (Implen, Westlake Village, CA, U.S.A.). Furthermore, cDNA was produced from 1 g of total RNA per sample utilizing a Sensi Fast cDNA synthesis kit (Bioline, Taunton, MA, U.S.A.). The newly synthesized cDNA was combined with a master mix (TaKaRa, Otsu, Japan) and appropriate target primers to investigate the tissues response to the induced wound: COL31 to assess collagen deposition, FGF-7 to evaluate wound re-epithelization, VEGF-A to evaluate angiogenesis, and TGF- to evaluate closure of wounds. Reactions were carried out on a Pikoreal system (Thermo Fischer Scientific, Waltham, MA, U.S.A.). Gene expression of the excised tissue at each time point was compared to the housekeeping gene glyceraldehyde 3-phosphate dehydrogenase (GAPDH). Primers utilized in gene expression analyses are depicted in Table 1. The results were normalized based on the GAPDH level. Each biological sample was replicated three times, and the findings were reported as the mean and the standard error.

**Table 1:** List of primer used in gene expression analysis

Gene	Primer sequence	Accession number
GAPDH	F: GGAGTAAACGGATTTGGCC	XM_014834961
	R:CATGGGTGGAATCATACTGAAA	
TGF-β1	F:TAATTCCTGGCGCTACCTCA	HM569606
	R:CATGAGGAGCAGGAAGGGT	
FGF-7	F: GACAGTGGCAGTTGGAATTGT	NM_001163883
	R: CAACAAACATTTCTCCTCCACTG	
VEGF	F: TCATTTCTCCAGGGTTTACCCT	XM_014837457
	R:ATTTGGGGGAGTAGAAGAGCAA	
COL3 α1	F: TTCCTGGGAGAAATGGTGACC	XM_014852914
	R:GGAGAATAGTTCTGACCACCAGT	

### Statistical analysis

Statistical analysis was conducted utilizing the 5.1 version of the statistical software (Graphpad Prism). Based on the outcome of the Kolmogorov–Smirnov test, the normal distribution was evaluated. The nonparametric Kruskal–Wallis test was employed at different time periods to analyze statistical differences between the various therapies and time. The results were presented considering the time; time × treatment interaction effect. The one-way ANOVA was performed at each time there was a significant effect. Results were considered significant when P < 0.05. Data were presented as the mean ± standard deviation (SD). Gene expression analysis results were expressed as mean with standard error of the mean. Two-way ANOVA was employed for comparison between means at different time points. Duncanʼs posthoc test was used to determine a significant difference between means at a p-value of 0.05.

## Results

### Macroscopic and clinical findings

At the 3rd week post wound induction ABP- treated wounds demonstrated a transparent and thin exudate compared to a moderate amount in the control wounds. In the 5th week post wound induction, the exudate completely subsided in the ABP-treated wounds but persisted in the control wounds until the 9th week. One week post wound induction, the ABP dressings remained well-fixed, stable, and covered the wound beds in ABP wounds. The ABP dressings had a glistening appearance, with the exception of the membrane’s free part and its margins overlapping the wound boundaries that exhibited brown coloration (Fig. 1B). In the 3rd week, the ABP- treated wounds were filled with normal healthy granulation tissue.

**Fig. 2:**
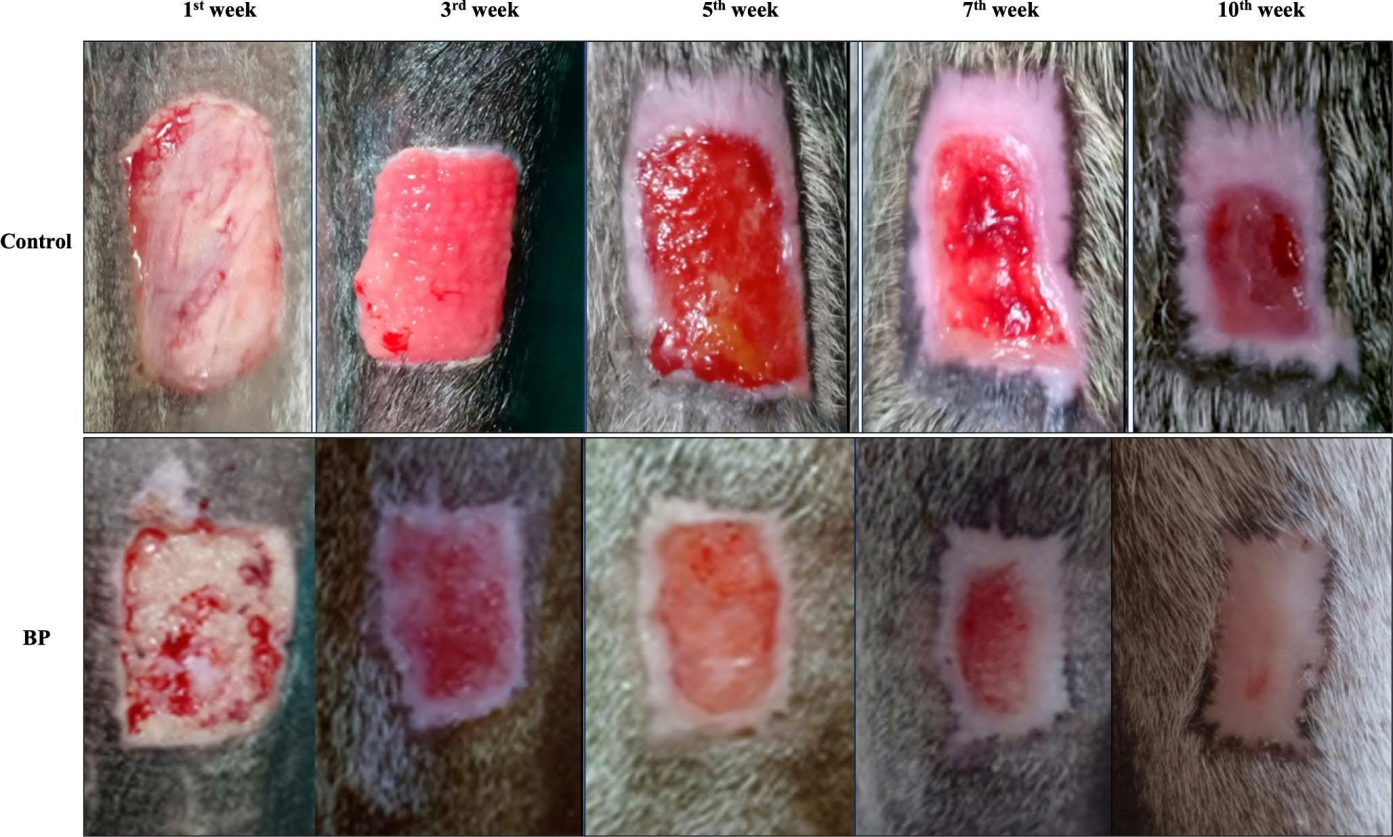
Progress of wound healing in acellular bovine pericardium (ABP) treated wounds and control wounds at different weeks

On the contrary, the control wounds showed a slight increase above skin level (Fig. 2). Significantly decreased wound dimensions were observed in the first week in the ABP-treated wounds compared to the control wounds (7.2 ± 0.3, 6.3 ± 0.1 cm^2^, respectively). Rates of wound contraction, epithelization, and healing were increased in the ABP- treated wounds compared to the control (Table 2). Overall, the interval days required for wound defect healing were significantly shorter (P < 0.05) in ABP-treated wounds (69.5 ± 1.6 days) compared with untreated wounds (86.3 ± 3.1 days). Signs of inflammation in the control wound were recorded and completely subsided by the completion of the second week, while it was not detected in the ABP wounds.

**Table 2:** Mean values and standard deviations of wound size (cm2), wound contraction (%), epithelization (%), healing (%) and granulation tissue formation score in control and BP treated during skin wound healing of distal limbs in donkeys

Criteria	Groups	Time post treatment (week)					
		1st	3rd	5th	7th	9th	10th
Wound size (cm^2^)	Control	7.2 ± 0.3^ A,a^	5.5 ± 0.08^ A,b^	4.4 ± 0.3^ A,b^	3.7 ± 0.2^ A,b^	3.2 ± 0.3^ A,b^	2.8 ± 0.2^ A,a^
	BP	6.3 ± 0.1^B,a^	4.5 ± 0.14 ^B,b^	2.9 ± 0.13 ^B,b^	2.3 ± 0.06 ^B,a^	1.4 ± 0.14^B,c^	1.1 ± 0.1^B,b^
Wound contraction (%)	Control	-20.7 ± 4.6 ^A,a^	8.7 ± 1.8 ^B,a^	27.3 ± 4.8^B,a^	39 ± 2.8 ^B,a^	47.5 ± 5.1^B,b^	53.3 ± 3.5 ^B,a^
	BP	-5.7 ± 1.6 ^B,b^	24.3 ± 2.4^ A.b^	51.8 ± 2.3^ A,b^	69 ± 2.4^ A,b^	77 ± 2.4^ A,b^	81.5 ± 1.8^ A,b^
Epithelization (%)	Control	0.00 ± 0.00	9.6 ± 1 ^A,a^	32.7 ± 1.8 ^A,s^	45.7 ± 1.9 ^A,a^	50.8 ± 1.9 ^A,a^	54.7 ± 1.8 ^A,a^
	BP	0.00 ± 0.00	28.7 ± 1.6^B,b^	57.6 ± 2.5^B,b^	74.7 ± 1.8^B,b^	85.6 ± 1.8^B,a^	89.7 ± 1.8^B,a^
Wound healing (%)	Control	-20.7 ± 4.6^B,e^	8.8 ± 1.6 ^B,a^	24.6 ± 1.8 ^B,a^	41.7 ± 1.6 ^B,b^	56.5 ± 1.8 ^B,a^	66.7 ± 1.7 ^B,a^
	BP	-5.7 ± 1.6^ A,e^	31.6 ± 2^ A,a^	46.7 ± 1.7 ^A,b^	65.7 ± 1.7 ^A,a^	81.6 ± 1.9 ^A,a^	85.8 ± 7 ^A,a^
Granulation tissue formation score	Control	1 ± 0^ A^	3.2 ± 0.8^ A^	2 ± 0.6^ A^	1.6 ± 0.5^ A^	1.5 ± 0.5^ A^	1.2 ± 0.4^ A^
	BP	1 ± 0^ A^	1.5 ± 0.5^B^	1.5 ± 0.5^ A^	1.3 ± 0.5^ A^	1.2 ± 0.4^ A^	1 ± 0^ A^

^a,b^ Small letter for different time point: means within the same raw carrying different letters are significantly different at (p < 0.05)

^A, B^ capital letters for different groups means within the same column carrying different letters are significantly different at (p < 0.05)

## Histopathological findings

**Fig. 3:**
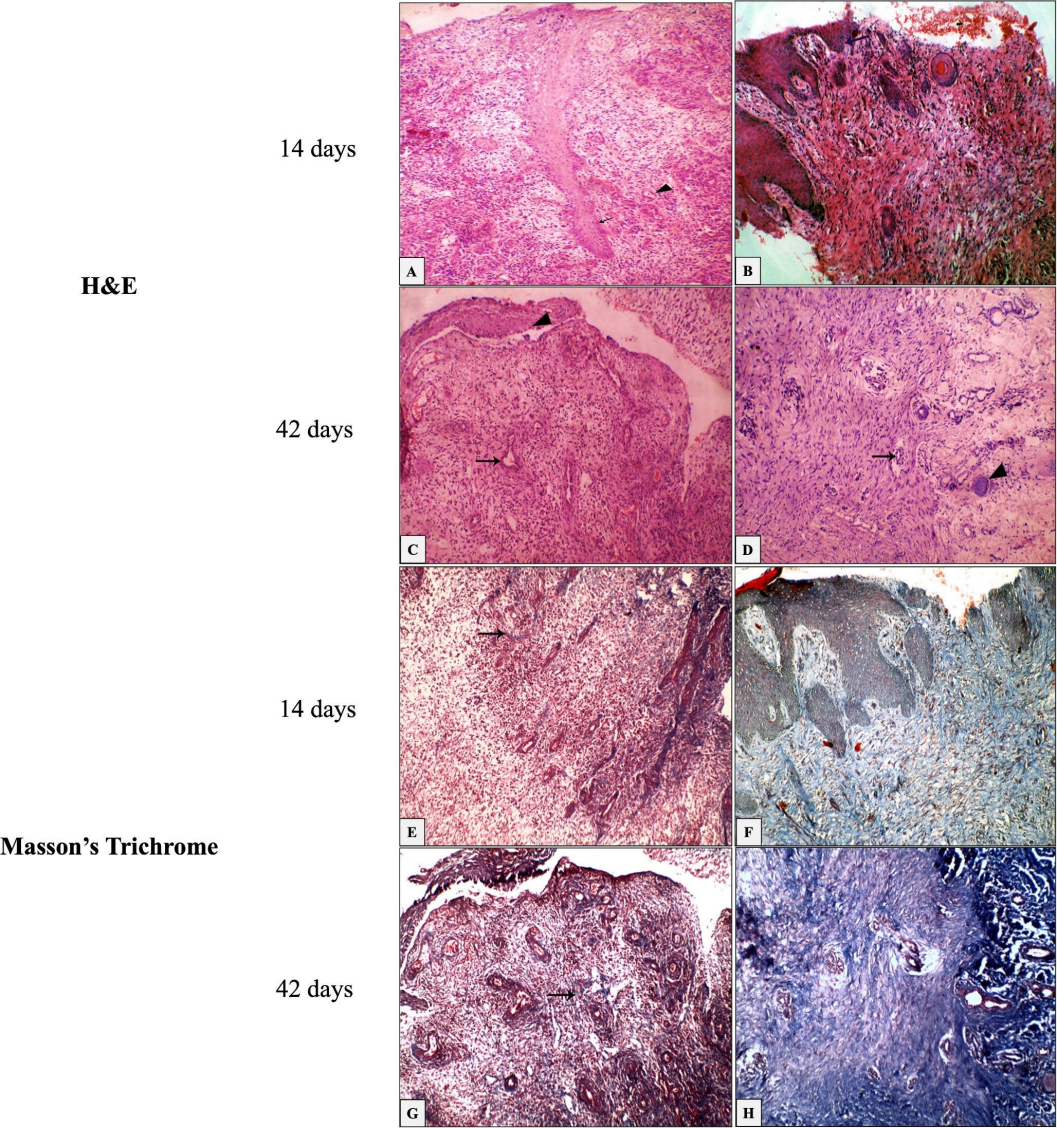
Histology of wound biopsies 14 and 42 days after treatment with H&E, 20 x (A-D) and Masson trichrome stain (E-H). (A) immature blood vessel (arrowhead), with frequent mitosis of epithelium (arrows); (B) active reepithelization with active mitosis in basal cell layer (arrows); (C) some mature blood vessels (arrow), mitosis with migration of epithelium on surface of wound (arrowhead); (D) well developed blood vessels (arrow) with synthesized collagen bundles arranged in normal wavy bundles along the dermis. Formation of hair follicular epithelium was noticed (arrow head). (E) in control wounds showed minimal fine scattered bluish stained collagen fibrils (arrow); (F) 14 days post injuries in ABP-treated wounds collagen bundles haphazardly arranged with epithelial forming islands inside it; (G) 42 days post injuries in control wounds less bluish stained collagen fibrils forming strands principally at perivascular (arrow); and (H) 42 days post injuries in ABP-treated wounds excess bluish collagen bundles in granulation tissue with parallel bundles

Histological analysis of ABP treatment revealed the formation and organization of new collagen and some well-ordered dermo-epidermal cell interventions, including fibroblasts, keratinocytes, macrophages, leukocytic cells, and neutrophils, as well as the endothelial cells of the blood-forming tissues, in addition to new collagen formation and organization. On days 14 and 42 after wound induction, collagen fibers in ABP-treated wounds were compactly structured, well-organized, and dispersed in a parallel pattern without segmentation, which did not occur in control wounds (Fig. 3 A-D). In contrast, Masson’s trichrome staining of the collagen fibers revealed the well-organized collagen fibers and the retention of collagen intensity in both conditions (Fig. 3E-H).

The histopathological assessment and semi-quantitative evaluation after wound induction revealed significant variations in wound healing progression between the ABP-treated wounds and control wounds (Table 3). There were more dermo-epidermal cell interventions in the ABP-treated wounds compared to the control wounds, such as fibroblasts, keratinocytes, leukocytic cells, neutrophils and macrophages, and endothelial cells of the blood-forming tissue, along with the new formation organized collagen fibers.

**Table 3:** Semi-quantitative evaluation of histological changes / structures during skin wound healing in control wounds and BP treated wounds

Treatment groups	Days	Epithelization	PMNL	Tissue macrophages	Fibroblasts	Neo-angiogenesis	New collagen
Control	14	0.3 ± 0.5^ A^	0.5 ± 0.5^ A^	0.5 ± 0.5^ A^	0.8 ± 0.4^ A^	0.8 ± 0.4^ A^	0.2 ± 0.4^ A^
ABP		1.2 ± 0.8^ A^	1.7 ± 0.5^B^	1.3 ± 0.8^ A^	1.8 ± 0.8^B^	2 ± 0.0^B^	1.2 ± 0.04^B^
Control	42	1.2 ± 0.4^ A^	0.8 ± 0.4^ A^	1 ± 0.0^ A^	1.7 ± 0.5^ A^	1.5 ± 0.5^ A^	0.7 ± 0.5^ A^
ABP		2.5 ± 0.5^B^	2.3 ± 0.5^B^	2.5 ± 0.5^B^	2.8 ± 0.4^B^	2.5 ± 0.5^B^	2.8 ± 0.4^B^

^A, B^ letters indicated the different between groups means within the same column carrying different letters are significantly different at (p < 0.05)

## Immunohistochemical analysis

**Fig. 4:**
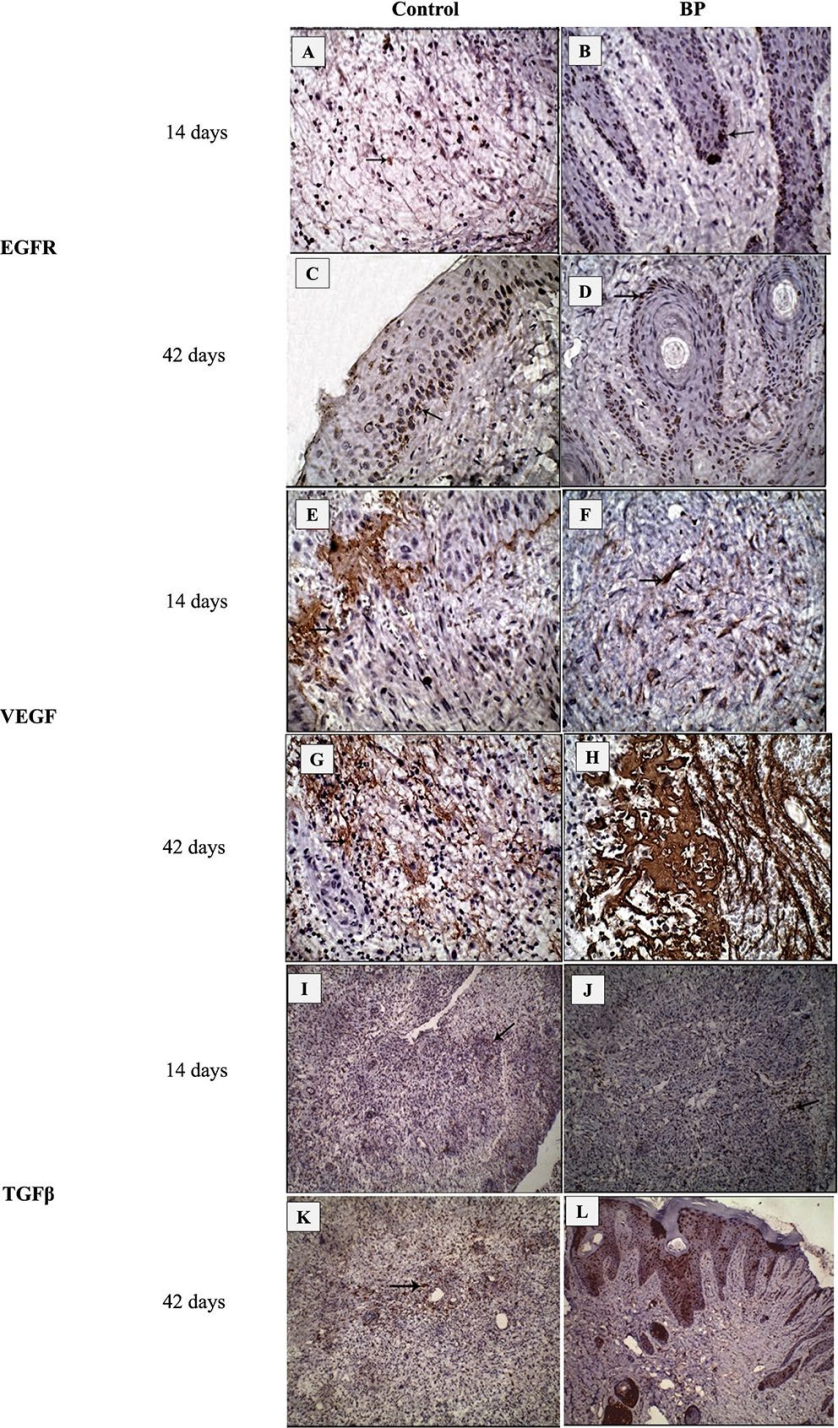
Immunohistochemical views at 14 and 42 days, EGFR (A-D), VEGF (E-H), TGFβ (I-L). Control (A, C), ABP (B, D) treated wounds showed a strong positive brown immunostaining for epithelial cells in ABP-treated wounds compared to control wounds. Control (E, G), ABP (F, H) treated wounds showed a strong positive brown immunostaining for newly formed angioblasts in ABP-treated wounds compared to control wounds. Control (E, G), ABP (F, H) treated wounds showed a strong positive brown immunostaining for fibroblasts in ABP-treated wounds compared to control wounds

Qualitative analysis of the wounds’ immunohistochemical views demonstrated a substantial immunostaining variation against TGFβ, VEGF, and EGFR stains between ABP-treated wounds and control wounds (Fig. 4). The anti-EGFR antibody immunohistochemical analysis displayed more intense and significantly higher staining in ABP than control wounds at different time points 14 and 42 days post wound induction (174.5 ± 4.5 and 218 ± 2.6 versus 43.8 ± 1.7 and 76.2 ± 3.1). VEGF in ABP-treated wound tissue at 14 and 42 days post wound induction was substantially enhanced and significantly increased compared to the control wounds (213 ± 9.5 and 258.5 ± 13.9 versus 120.5 ± 1.9 and 151.5 ± 6.3). The average count of cells immunostained for TGFβ at 14 and 42 days post wound induction demonstrated a substantial improvement in the ABP-treated wounds compared to control wounds (125.2 ± 3 and 146.3 ± 14.7 versus 25.5 ± 2.5 and 86 ± 3.7).

## Gene expression analysis

A relative expression of COL3α1, FGF-7, TGFβ1, and VEGF-A demonstrated a substantial upregulation in the ABP-treated wounds (P < 0.0001) in comparison with control wounds (3.3 ± 0.1, 5.5 ± 0.14, 2.45 ± 0.17, and 4.26 ± 0.13 versus 1 ± 0.06, 1 ± 0.04, 1 ± 0.08, and 1 ± 0.03 folds, respectively) at 14 days post wound induction. Additionally, at 42 days after wound induction, there was a significant increase (P < 0.0001) of COL3α1, FGF-7, TGFβ1, and VEGF-A expression in the ABP-treated wounds compared to the control wounds (5.2 ± 0.08, 8.29 ± 0.14, 4.67 ± 0.12, and 6.18 ± 0.17 versus 1 ± 0.1, 1 ± 0.2, 1 ± 0.05, and 1 ± 0.1 folds, respectively).

## Discussion

Many factors affect the healing of equine leg wounds: wound expansion, contraction, epithelization process, granulation tissue formation, and contamination (Dart et al. 2009). Moreover, bony prominence in this area, deep supportive tissue deficiency, and higher joint motion give equine distal limb wounds a long preparatory phase (Schwartz et al. 2002; Celeste et al. 2011). Therefore, it is important to improve wound healing in equines by increasing the contraction rate, promoting epithelialization, averting the exuberant granulation tissue formation, diminishing the scar tissue, ultimate cosmetic attendance, and enhancing the chances of a return to complete athletic effectiveness (Jørgensen et al. 2021). This study confirmed that, topical dressing of ABP on distal limb wounds in donkeys accelerated and potentiated their healing, especially if repeatedly applied for three times with one week interval.

ABP is a xenograft used in the current study as the first layer of limb wound dressing in ABP wounds. It is important to prepare it by decellularization, which is considered an essential step because BP has a potent antigen that triggers immunological reactions in the recipient, triggering graft rejection. Moreover, a decellularization protocol can effectively remove all cell components and nucleic residues (Gilbert et al. 2007). Furthermore, the organ and its extracellular matrix components retain all the essential signals for cell preservation (Fu et al. 2014). Mechanical cleaning of the ABP membrane using dry gauze was performed manually to remove all connective tissue and unwanted fat from the ABP and to make a rough surface. The biomaterial’s roughness modulates the tissues’ biological response to the implants (Mondalek et al. 2008). It plays a crucial role in the adhesion and cellular behavior (dos Santos et al. 2017) and in avoiding immune rejection and minimizing inflammatory reaction after the application into the wound (Gilbert et al. 2007; AL-Bayati and Hameed 2018).

Before the application of the ABP fragment, it was cut 0.5 cm broader than the wound to maintain the moist environment of the wound as much as possible and rinsed in saline and then in penicillin antibiotic solution for 15 min for rehydration and to increase the resistance against infection (Bellenzani et al. 1998).

No signs of inflammation were noted in the post-wound induction phase in the ABP-treated group. On the contrary, signs of inflammation were noted in the control wounds for 11 days. This difference can be attributed to the cellular inert state of the ABP (Gilbert et al. 2007; Guerra et al. 2016). Similarly, the absence of immune rejection signs in the ABP-treated wounds may be related to decellularization. This result agreed with (Anson and Marchand 1996), who studied the effect of ABP as a dural graft material in human patients undergoing spinal surgeries.

In the current study, we used a single dose of systemic antibiotics with no NSAID to avoid fog wound healing evaluation (Bellenzani et al. 1998; Ibrahim et al. 2020; Duddy et al. 2022).

(Afifah et al. 2019) illustrated that the process of wound healing does not induce pain. On the contrary, (Gültiken et al. 2022) mentioned that postoperative pain might cause postoperative complications such as prolonged recovery time or delayed wound healing, therefore in this study, butorphanol was administrated for 3 days post-wound induction.

In both groups, there were no signs of infection or contamination that potentially may be contributed to the systemic administration of antimicrobials and serial dressing. Moreover, the ABP dressings function as biological scaffolding that showed remarkable resistance to bacterial infection (Jernigan et al., 2004; Shell IV et al., 2005) even in clinical applications with a high risk for bacterial contamination (Kim et al. 2005). These properties were evidenced by the findings of (Holtom et al. 2004), who illustrated that the antibacterial activity linked to small intestine submucosa ECM is not a feature of the intact ECM but rather a trait of the ECM degradation products. Furthermore, covering the wound gaps by normal dressing or ABP sheet has emulated the favorable properties of the scab, wound protection, and preserving a favorable environment for healing. (Hanson 2008) mentioned that the scab should be able to stop bleeding, protect the wound, inhibit bacterial infections, and help tissue regeneration.

Nevertheless, in the case of the ABP sheet, the minimal granulation of tissue formation benefited the start of the healing process. These results were also recorded by (Reyes et al. 2010) during the closure of the bronchopleural fistula. The scaffold was suppressed and degraded by host cells and induced the formation of site-specific functional host tissue (Gilbert et al. 2007).

One week after wound induction in the current study, the ABP dressings shrunk in size and changed their margin’s color to brown, called a degradation process. (Swinehart and Badylak 2016) reported a function of the degradation process as it plays an essential role in the healing process by releasing growth factors sequestered within the matrix. This process was started in the first week, so re-dressings of ABP dressings were performed every week. Decreasing the wound dressing times can relieve stress and pain correlated with dressing change. These findings agree with other studies (AL-Bayati and Hameed 2018; Ibrahim et al. 2020) that evaluated the ABP and other biological dressings on cutaneous wound healing in rabbits and donkeys.

The surgically induced distal limb wounds were maximized in size one week after operation in the control wounds (7.2 ± 0.3) and treated group (6.3 ± 0.1), then declined throughout the whole study procedures. This enlargement may be attributed to greater mobility and the skin tension forces that retract the skin edges and cause wound expansion. These observations were similar to the results reported by (Schwartz et al. 2002). They noticed that the balance between collagen synthesis and degradation, especially in the distal aspect of the distal limb wounds, was a crucial step in avoiding exuberant granulation tissue and delayed wound healing. In the current study, using ABP as a wound dressing resulted in faster healing rates (85.8%) than control wounds (66.7%). This suggests that it is vital to prepare the BP to maintain the ECM responsible for releasing growth factors and promoting angiogenesis and other cellular responses in the local tissue. These findings are compatible with those of (Reing et al. 2010), who found that the processing method affects the porcine dermal ECM. Consequently, appropriate processing steps to maintain ECM beneficial effects in biological scaffolds must be carefully selected (Choi et al. 2013).

Increased epithelialization rate in ABP-treated wounds compared to the control wounds can be attributable to the keratinocyte growth factor, a crucial growth factor that encourages wound epithelialization and stimulates keratinocytes differentiation into different types of epithelial cells. These results agreed with (Badylak et al. 2008), who demonstrated that the composition and organization of extracellular matrix closely approximate the tissue to be replaced.

Wound contraction was substantially increased in ABP-treated wounds than in the control wounds. This result can be attributable to the fibrous connective tissues’ high deposition and fibroblast infiltration, in addition to the action of myofibroblasts that are crucial for wound margin’s centripetal movement (AL-Bayati and Hameed 2018).

Furthermore, the fixation of the ABP sheet to the wound margins did not impede contraction. However, it can hinder expansion, which aligns with (Bellenzani et al. 1998), who recorded a beneficial effect of the pericardium on granulation tissue when used pericardium dressing on distal limb wounds in horses. Histopathologically, the ABP sheet efficiently interacted with the wounds and provided protection, adhesion, and a moist healing environment. The benefits of human placenta-derived ECM that contain bioactive molecules have been described as full-thickness skin wound healing in the rat model (Choi et al. 2013). The enhancement of the epidermal epithelialization in ABP wounds was associated with marked proliferative actions and high mitotic activities in epidermal and dermal layers, also reported by (Roberts et al. 1988).

Cell quantification for VEGF was significantly higher in the ABP- treated wounds showing that ABP is a continuous source of VEGF, which significantly contributes to the healing of wounded tissue, as reported by (Reing et al. 2010). Additionally, the occlusive nature of the wound dressing maintains the wound’s moisture and enhances the healing process (Tan et al. 2019). In addition, the formation of new capillaries across freshly produced granulation tissue stimulated the healing process, consistent with reported observations in humans and rats (Gilbert et al. 2007; Van Rijn et al. 2016).

Cell quantification immunostained for VEGF demonstrated that ABP’s results were significantly higher. ABP is considered a source of VEGF that significantly contributes to the healing of damaged tissues, as reported by (Wang et al. 2011; Miron et al. 2017).

Moreover, cell quantification immunostained for EGFR demonstrated ABP’s crucial role in promoting the epithelization process that is fundamental to the healing process. These results showed that the scaffolds were successfully integrated into wounds. The closure was achieved through re-epithelialization by endogenous wound keratinocytes (Nurmaulinda et al. 2021), as EGFR greatly increases the speed of macroscopic healing in tissue treated with PRP than in control wounds. Additionally, cell quantification immunostained for TGFβ demonstrated ABP contribution as a source of TGF in promoting angiogenesis, epithelial cell proliferation, and the organization of wound sites, as referred by (Wasterlain et al. 2016).

The gene expression of donkeys in the present study exposed to ABP that was applied to distal limb wounds demonstrated substantially elevated COL3α1, TGFβ1, VEGF-A, and FGF-7 gene expression at all time points compared to control wounds. Similar findings were reported by (Kinbara et al. 2002; Komi-Kuramochi et al. 2005; Hatakeyama et al. 2007; Schnabel et al. 2007) that can be attributed to the number of growth factors provided by ABP dressings. These findings agree with (da Fontoura Pereira et al. 2019), who studied the effect of platelet-rich plasm and its growth factors on equine distal limb wounds.

Several limitations were associated with the present study, some of which were related to the experimental model. For example, the wounds were created surgically rather than traumatically and did not invade the underlying subcutaneous or deeper tissues. Additionally, in contrast to naturally occurring wounds, the wounds in the current study were dressed immediately with little opportunity for contamination. The clinically relevant dressing of distal limb wounds three times with ABP dressings accelerated wound healing in donkeys. However, it is possible that naturally occurring, chronic, or nonhealing wounds would respond differently, and permanent occlusion is inappropriate for the treatment of exuding wounds.

## Conclusion

To our knowledge, this study presents the first report on using ABP as a biological scaffold for distal skin limb wounds in donkeys. Our data demonstrated that using ABP dressing is a safe and effective treatment for cutaneous distal limb wounds in donkeys.

## Data Availability

All data generated or analyzed during this study are included in this article.

## Abbreviations

**ECM**: Extracellular matrix
**ABP**: Acellular Bovine pericardium
**VEGF**: Vascular endothelial growth factor
**COL3α1**: Collagen type 3α1
**FGF-7**: Fibroblast growth factor-7
**TGFβ1**: Transform growth factor β1
**EGFR**: Epidermal growth factor receptor
**PBS**: Phosphate buffered saline
**GAPDH**: Glyceraldehyde 3-phosphate dehydrogenase
